# Thermal Imaging Detection System: A Case Study for Indoor Environments

**DOI:** 10.3390/s23187822

**Published:** 2023-09-12

**Authors:** Martin Drahanský, Michal Charvát, Ivo Macek, Jitka Mohelníková

**Affiliations:** 1 TrendBit s.r.o., 616 00 Brno, Czech Republic; 2 Faculty of Civil Engineering, Brno University of Technology, 612 00 Brno, Czech Republic; charmiv@seznam.cz (M.C.); mohelnikova.j@fce.vutbr.cz (J.M.); 3 National Museum Prague, 110 00 Prague, Czech Republic; ivo.macek@nm.cz

**Keywords:** artificial intelligence, infrared imaging, infrared sensors, localization and mapping, neural networks, building security management

## Abstract

Currently, there is an increasing need for reliable mechanisms for automatically detecting and localizing people—from performing a people-flow analysis in museums and controlling smart homes to guarding hazardous areas like railway platforms. A method for detecting people using FLIR Lepton 3.5 thermal cameras and Raspberry Pi 3B+ computers was developed. The method creates a control and capture library for the Lepton 3.5 and a new person-detection technique that uses the state-of-the-art YOLO (You Only Look Once) real-time object detector based on deep neural networks. A thermal unit with an automated configuration using Ansible encapsulated in a custom 3D-printed enclosure was used. The unit has applications in simple thermal detection based on the modeling of complex scenes with polygonal boundaries and multiple thermal camera monitoring. An easily deployable person-detection and -localization system based on thermal imaging that supports multiple cameras and can serve as an input for other systems that take actions by knowing the positions of people in monitored environments was created. The thermal detection system was tested on a people-flow analysis performed in the Czech National Museum in Prague. The contribution of the presented method is the development of a small and simple detection system that is easily mountable with wide indoor as well as outdoor applications. The novelty of the system is in the utilization of the YOLO model for thermal data.

## 1. Introduction

### 1.1. Topicality of the Problem

The human detection and localization problem represents one of the contemporary topical research tasks [1,2,3,4,5]. Detection technology has found applications in many areas of everyday life. It is often used for queue management in shops, people-flow analyses in museums and exhibition places, marketing for determining the best product placement, and people-detecting mechanisms in smart homes with controlled indoor environments. They can help to ensure safety in heavy-machinery workplaces of industry halls or at railway stations by guarding hazard zones.

The presented research focuses on a system of people-detection using thermal imaging. Thermal imaging systems are performed on the basis of infrared radiation detection. Thermal imaging sensors can obtain an image of the human body from thermal emissions in the spectral range of long wavelength infrared radiation, from 8 to 14 μm. These systems do not require any illumination from sunlight or moonlight or the use of an infrared illuminator [6,7].

### 1.2. Related Technologies

The following overview of the detection technologies shows the current possibilities of systems used for people-detection. The methods behind these detection algorithms are summarized below.

#### 1.2.1. Detection Technologies

Some of the current technologies being used for counting, detecting, and locating people are as follows:

- Infrared/laser beam interruption: Such a system counts people passing through a narrow passage, for example, a doorway. A transmitter device is installed on one side of the passage, and a receiver is installed on the other [8]. The two devices are connected together and form an invisible barrier of light. When an object breaks the connection between the transmitter and the receiver, the system registers a plus-one count.
- Light Detection and Ranging (LIDAR) is a method for measuring distances (ranges) by illuminating the target with a laser light and measuring the reflection with a sensor. It consists of a single sensor, usually placed above a passage. The sensor acts as a transmitter and a receiver at the same time. The device casts laser beams in several directions and precisely measures the time required for each reflected beam to return to the sensor. It is possible to calculate the distance of each laser beam and, therefore, to create a depth map revealing objects in its field of view [9,10].
- GPS/Wi-Fi/Bluetooth tracking is a method of tracking people using wireless technologies such as Wi-Fi or Bluetooth. These smart technologies make it easy to triangulate the position of people and to create movement maps [11].
- Projecting structured light: Such a system usually consists of two parts—a camera and a projector. The projector casts structured light on the scene. The structured light is usually a horizontal black-and-white-lined pattern or a checkerboard pattern. The camera is then used to view the scene, and by analyzing deformations in the projected pattern, a depth map is constructed. This method is not directly used to detect or locate people, but is more often used for obtaining 3D models of relatively small objects or continuous depth maps [12].
- Three-dimensional stereo video analysis: Such a system consists of two precisely calibrated cameras viewing a scene. The technology is somewhat similar to human vision—two eyes viewing a scene, with the brain extracting depth information from differences in the two shifted images.
- Monocular video analysis is quite similar to 3D stereoscopic vision; however, for monocular video analysis, no depth map is used, as the monocular video analysis uses only a single camera [13].

#### 1.2.2. Detection Algorithms

People-detection is a task of placing boxes around objects in an image. The object-detection problem has been studied since 1960. Facial detectors appeared about twenty years ago. Then, detection algorithms were developed. The Viola–Jones algorithm [14], released in 2001, uses hand-coded features that are fed into a *support vector machine* (SVM) classifier. The hand-coded features for facial detection are the positions of the eyes, nose, and mouth and their relation with respect to each other. The algorithm performs correctly when detecting faces, matched with the hand-coded features; however, a problem with detecting rotated faces or faces in any other orientation has appeared [15].

In 2005, a new detection algorithm was released. A *histogram of oriented gradients* (HOG) [16] is used as a feature descriptor, where each pixel of the image is replaced by a gradient showing the direction of decreasing pixel intensity with respect to the surrounding pixels. The image is then divided into squares, and all gradients inside each square are merged into a single most-dominant gradient. During this process, an image is replaced by a simple representation of the essence of the image using gradients. The algorithm uses a similarity metric to determine how close an image is to the object we are looking for by comparing their gradient patterns [17].

Progress came in 2012 when the CNN-based system AlexNet was developed [18]. Convolutional neural networks have been known since the 1990s. Recent increases in processing power and the amount of data are because of the neural network potential. The convolutional neural network, in essence, learns the feature descriptors on its own during the training process, as opposed to the previous two methods in which they had to be crafted by hand.

These three algorithms, as described, correspond more to object classifiers, meaning that they can tell what the object in an image is if there is nothing but one object in it. It cannot detect and classify multiple objects in a single image. This has, however, been proven to be possible by repurposing any image classifier. The classifier can sequentially classify every part of an image through a sliding window, and detections with the highest confidence score, in that case, represent the output of the detector. This approach is, however, extremely computationally expensive. In 2014, the R-CNN object detector [19] was released, followed by the Fast RCNN [20] and Faster R-CNN [21] in 2015. They were used for the selective search technique instead of a sliding window to create sets of bounding boxes that were later completed in the classifier to cut down the number of operations.

In 2015, the new YOLO algorithm was introduced. You Only Look Once (YOLO) is a state-of-the-art real-time object-detection system [22]. When compared with all other detectors, it takes a completely different approach. Instead of repurposing an image classifier and using it to classify different regions in the image, this approach uses a neural network that takes an image to the input and, in one single pass, outputs the regions with detected classes and scores for every region. This new approach is required to redefine the parametrization of the object detection. Every image is split into a grid, where each grid-cell is responsible for predicting several bounding boxes and confidence scores for each bounding box, saying how sure the detector is that a certain bounding box actually contains an object and, if there is an object, what kind of object it is.

In 2016, the second version of YOLO was released [23], featuring important improvements that increased its overall accuracy. The detector finished training on images with higher resolutions and changed the way of representing bounding boxes. YOLOv2 uses dimension clusters to represent bounding boxes. Using unsupervised learning, the creators extracted the five most common shapes and sizes of bounding boxes occurring in the VOC 2007 image dataset, and used them as templates for bounding boxes that each cell in the YOLOv2 detector can detect. YOLOv2 also uses multi-scale training, meaning that the input image size is not fixed throughout the training process, but changes on the fly, resulting in a more robust detector, as it works better on differently sized images.

The third version, YOLOv3 [24], brought even more improvements in 2018. One of them is the support for multiple labels. More importantly, YOLOv3 uses a new backbone (or feature extractor part of the network), Darknet-53. The network has 53 convolutional layers with short-cut connections, allowing for the extraction of finer-grained information from the image. This significantly improves the detection of accuracy of small objects. Unlike the previous versions, YOLOv3 makes bounding box predictions at three different scales, improving the accuracy of the detector.

The fourth version of YOLO [25] was released in 2020. This version promised even better accuracy and speed, effectively dominating every other solution in the field of real-time object detection. For this version, the creators performed an ablation study to test and select the most effective training optimization methods, which lead to improvements in accuracy with minimum additional computational cost.

The tested methods were mostly data augmentation techniques that could potentially increase the descriptive power of the feature-extracting part of the network. Some of the data augmentation methods are the following: edge map, flip, rotate, detexture, cutmix, mosaic, dropblock regularization, and so on. A new activation function has been tested, as well as other specialized techniques like cross-stage partial connections or multi-input weighted residual connections. The optimizations also covered selecting the optimal hyperparameters of the model, like the number of training steps, batch size, learning rate, momentum, weight decay, or minibatch size. YOLOv4 is superior to all other object detectors in terms of both its speed and accuracy.

## 2. Materials and Methods

The presented system is focused on people-detection applications. Using a thermal imaging camera module to solve the problem of people-detection, it belongs to the monocular video analysis section and brings several advantages when compared with other approaches. Only a single camera is needed, so there is no need for extremely precise hardware calibration of the system, as with the stereo vision or structured-light projection.

It is possible to detect and also locate individuals, in contrast with infrared/laser beam or light travel techniques, which can only count objects entering and leaving an area. Advantage of the system is in privacy. Facial recognition is not provided. This makes this approach more suitable for places where privacy plays an important role, e.g., at workplaces or homes. The system is not influenced by light conditions. Disadvantage is in infrared radiation reflections.

Thermal images are not dependent on lighting conditions of the scene, which makes the system very effective during the night. The largest disadvantage of monocular image analysis is the missing depth dimension. The lower camera resolution with the missing depth dimension causes locations of the detected objects to be only a rough approximate, as the missing dimension has to be estimated based on some assumption, like if the object is touching the ground. The idea of the detection and localization of people due to the thermal camera is to capture a thermal image, detect objects corresponding to people in the image, and estimate locations of each object in a model of the scene using the perspective projection.

The method of detection of people using thermal imaging camera deals with utilizing a single thermal-camera module, a single-board computer, and image processing. Using a small thermal camera module eliminates the possibility of person and/or face recognition while preserving the functionality of detecting and even locating people. The solution to the problem of people-detection based on thermal imaging is, therefore, a viable option for places where privacy plays an important role.

This method was developed within the frame of a research project. Main tasks of the project were:

- Thermal capture system composed of a Lepton 3.5 camera and a Raspberry Pi 3B+ single-board computer;
- C++ capture library, allowing one to read thermal images from the camera in both raw format and false color over SPI interface;
- Python control script, allowing one to issue commands to the camera over I^2^C in order to change a color palette, format, control automatic gain (AGC), perform flat-field correction (FFC), and other functions;
- Python scripts for person-detection and single-camera rectangular scene abstraction, allowing for reverse-projecting image points of detected people into a 3D scene model.

### 2.1. Thermal Capture Unit

**Figure 1:**
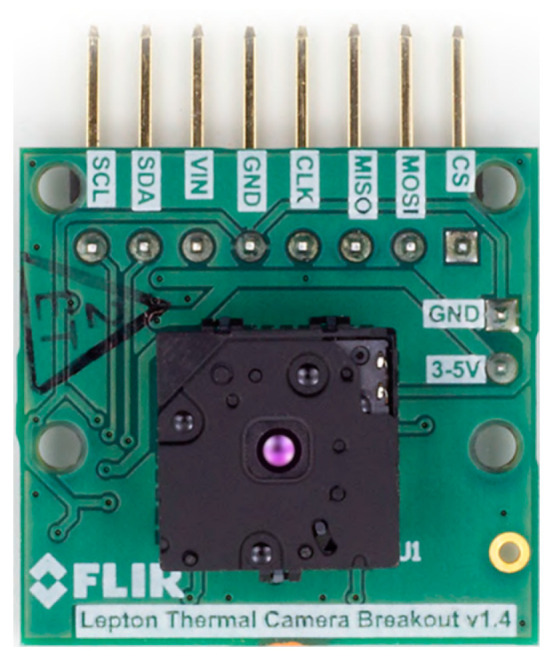
Lepton 3/3.5 with breakout board [27].

The thermal capture unit, which can be used for standalone or remote capture of thermal data, was completed. The unit can be placed anywhere with electric and network connection and consists of FLIR’s Lepton 3.5 [26] thermal camera module (Lepton 3/3.5 with breakout board is shown in Figure 1) and a custom PCB with a circuit controlling the thermal module and the Raspberry Pi 3B+ single-board computer, which communicates directly with the camera. All these three parts are enclosed in a custom-designed 3D-printed enclosure box.

The thermal unit uses Raspbian Buster Lite, which is a minimal operating system with only 435 MB in size. Since it is the minimal system, it is necessary to install all dependencies and libraries manually. The exact same steps would have to be performed on every thermal unit and repeated for every new unit. Therefore, it only makes sense to use a tool to automate the steps of preparing the environment on thermal units for running the detection and localization system.

**Figure 2:**
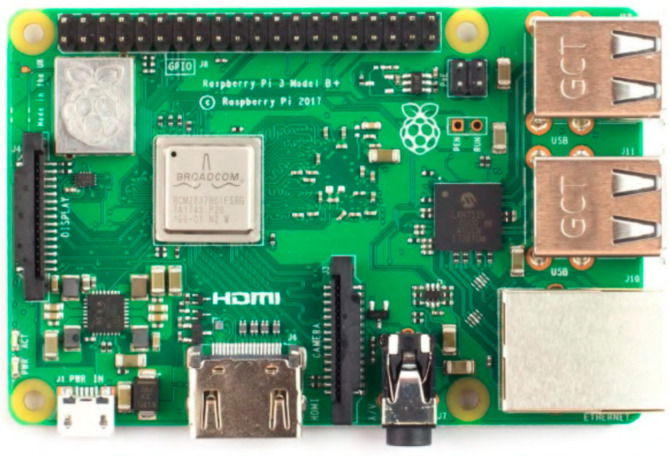
Raspberry Pi 3B+ single-board computer [28].

The Raspberry Pi 3B+ computer is used to directly communicate with the thermal camera, and is therefore the “brain” of the thermal unit. The Raspberry Pi 3B+ is the last revision of the third generation single-board computer. It is a low-cost, credit-card sized computer capable of performing everything one might expect from a regular desktop computer. The Raspberry Pi runs a Debian-based operating system, Raspbian Bustler Lite. The computer has a built-in hardware support for SPI, I^2^C, UART, Bluetooth, and Wi-Fi communication. In general, input/output (GPIO) pins are also extremely important for interfacing with other electronic devices. The Raspberry Pi 3B+ single-board computer is shown in Figure 2.

The Ansible [29] (software intended to do provisioning, configuration management, and application-deployment) was used by us for the automatic deployment. It is an agentless tool which temporarily connects to its targets via ssh to perform tasks specified in so-called Ansible playbooks.

A configuration on an SD card is due to writing the Raspbian Bustler Lite image to the SD card using, for example, the balenaEtcher tool—free open-source utility for creating live SD cards and USB flash drives [30], it can be inserted into the Raspberry Pi. The computer should boot up and connect to the network according to the configuration. After that, the Ansible can take over. From the master computer, the thermal units are configured by running the Ansible playbook. The thermal unit is ready to operate. In order to communicate with the camera using SPI and I2C hardware modules on the Raspberry Pi, they need to be enabled at the kernel level.

The v4l2lepton3 library is the main software part. It is a library that takes care of controlling the camera and retrieving thermal video feed from it. It was designed and implemented from scratch. The library consists of two parts: the C++ application for thermal video manipulation and a Python3 package for the camera control and single-frame manipulation. The control software is implemented in the v4l2lepton3.control Python3 module as a part of the v4l2lepton3 Python3 package available in the git repository. In the implementation, each command has exactly one definition, which automatically generates allowed methods and contains a translation map for each option that the command can set. For people-detection, the real YOLO detector is used based on DNN module on thermal dataset. The DNN module implements forward pass (inferencing) with deep networks, and is pre-trained using deep learning frameworks like Darknet.

The Lepton camera sends video frames over the SPI interface on 20 MHz, which implies that the length of wires connecting the Lepton camera to the Raspberry Pi needs to be as short as possible—maximum of 20 cm in order to provide a stable connection without interference and transmission errors. This condition enforces the need for the Lepton camera and the Raspberry Pi to be physically close to each other. Together, they form a thermal unit.

The camera contains a sensor sensitive to long-wavelength infrared radiation in spectral range from 8 to 14 μm [31]. The camera module is smaller than a dime and provides images with decent resolution of 160 by 120 pixels. The effective frame rate of the camera is only 8.7 Hz. The camera only requires a low voltage supply and has a small power consumption of about 160 mW. For better manipulation with the camera module, a breakout board was used. The power supply provides the camera module with three voltages: 1.2, 2.8, and 2.8–3.1 V. The breakout board also supplies the camera with the master clock signal. The camera uses two interfaces for the communication:

- SPI, for transferring the video frames from the camera to the SPI master device;
- I^2^C, for receiving control commands from the I^2^C master device.

In order to make the whole thermal unit transferable, protected, and professionally looking for quick demonstrations or real-life deployment, an enclosure has been designed to fit and mount all of its components—the Raspberry Pi 3B+, the custom camera switch circuit board, and the Lepton 3.5 camera. The thermal unit case is composed of two parts—an enclosure box for the Raspberry Pi with the power switch and a camera chassis that is mounted to the top of the first part with a bit of slack that allows the camera chassis to be moved along the horizontal axis.

The enclosure box was designed in Sketchup15 software and exported into.stl format for 3D-printing. The Lepton 3.5 camera chassis model was created using the official Lepton 15Sketchup—Trimble design software [32]. Breakout board [27] was taken from portal Thingiverse [33].

**Figure 3:**
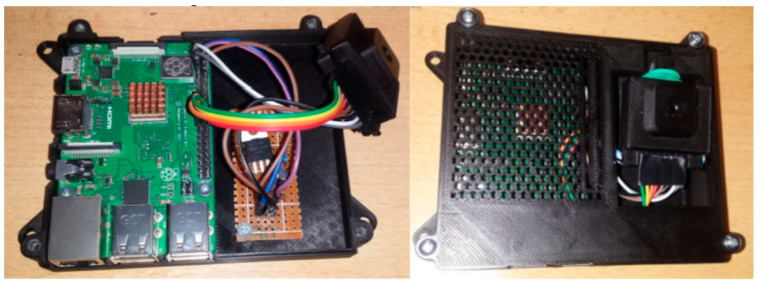
Finished thermal unit with Raspberry Pi 3B+, custom power switch, and the Lepton 3.5 thermal camera in the chassis.

Two pins were added in the lower part of the back side of the chassis to serve as pivot points around which the camera could move. The camera chassis with the Lepton camera is inserted into the hole on the right side of the top piece of the enclosure. The SPI and I^2^C interfaces are connected directly to the Raspberry Pi via approximately 10 cm long jumper wires. Two power wires are connected to the custom PCB with the switch, which is mounted in the enclosure underneath the camera, right next to the Raspberry Pi. From the custom PCB, there are three wires going to the Raspberry Pi directly—to GND, 5 V, and a virtual GPIO-15 pin. An assembled thermal unit is shown in Figure 3.

### 2.2. Capture and Control Library

The v4l2lepton3 library is the main software part of the project. It is a library that takes care of controlling the camera and retrieving thermal video feed from it. The library consists of two parts: the C++ application for thermal video manipulation and a Python3 package for camera control and single-frame manipulation. The Lepton camera provides a command and control interface (CCI) via a two-wire interface almost identical to that of I^2^C, with the only difference being that all transactions must be 16 bits in length. All Lepton’s registers are 16 bits wide.

Lepton camera offers 4 control registers and 16 data registers, which are all 16 bits wide and are used by the host (master) device to issue commands to the camera. A command is issued by writing and reading particular registers in the camera via I2C. The exact process is described in the CCI documentation [34]. For implementation, each command has exactly one definition, which automatically generates allowed methods and contains a translation map for each option that the command can set.

**Figure 4:**
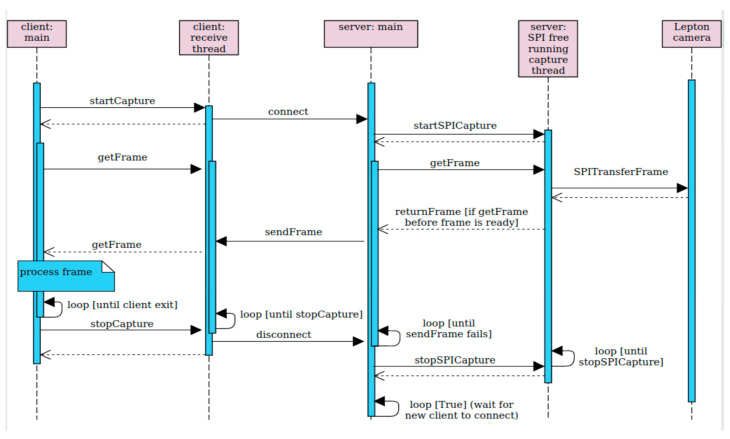
Sequential graph of the server–client model.

The implementation of the capture software follows the server–client model. It is written in C++ and forms a single process that runs on the Raspberry Pi computer to achieve maximum speed. The thermal unit behaves like a synchronous server. Its server process listens on a port and waits for a client to connect. Once a client is connected, it initializes the SPI interface and starts pulling frames from the camera and sending them over the open socket. When the connection to a client is lost, the thermal unit stops communicating with the Lepton camera and starts listening for another client. The sequential graph of the server–client model is presented in Figure 4.

The connection between the server and the client is realized using a TCP connection. The TCP transport protocol has been chosen because it ensures in-order delivery of every packet. If packets get lost or arrive out of order, it would not be possible to assure proper reconstruction of each frame. The server is sending the data frame by frame, pixel by pixel. The client keeps receiving bytes until 160 × 120 × 2 bytes are obtained. From these data, the client reconstructs the thermal frame in its raw format (Y16). Since the stream may be compressed by the zlib stream compressor, it is important to receive the whole frame and then decompress it.

### 2.3. Scene Reconstruction

The scene reconstruction and point protection from the camera to the 3D scene represents projecting objects from the image to the 3D scene model. In order to approximate coordinates of an image object in world space, it is necessary to understand the camera’s location and orientation in space. Knowing the pose of the camera allows us to reconstruct the 3D scene and display the camera and detected objects in it.

The implemented scene abstraction and the problem of computation of correspondence between image coordinates of a bounding box and 3D coordinates in the scene model was solved. The detection process yields bounding boxes around detected people represented by image coordinates [35,36,37,38,39,40,41,42,43,44]. The next step is to create an abstraction of the environment monitored by the camera, and then translate each detected object into an approximate location in the model of the environment.

In order to approximate coordinates of an image object in space, it is necessary to understand the camera’s location and orientation. The pose of the camera allows one to reconstruct the 3D scene and display the camera and detected objects. The camera-pose estimation problem is often referred as the Perspective-n-point problem (PnP).

The PnP is a problem of estimating the pose of a calibrated camera. By pose, the camera position and orientation is determined with respect to another coordinate system. The camera pose can be determined via rotation matrix and translation vector. Solving the PnP problem requires pairs of corresponding 3D to 2D mapping points [37,40,44,45]. Given those mapping points, estimating the pose is a matter of solving a system of linear equations. At least four pairs of points are required to find a solution. It represents a scene-abstraction script, allowing one to model a rectangular scene with a single camera and the reverse-project detected people into it.

The PnP problem can be expressed by Equation (1), which comes from the perspective projection (world to screen or world to image transformation):

(1) $$P_{i}=K\left[R|t\right]P_{w}$$

where

**P**_**i**_ is an image point (2D);
**K** is a matrix of intrinsic camera parameters;
**R** is a rotation matrix;
**t** is a translation vector;
**P_w_** is a world point (3D).

The expanded form of Equation (1) can be found in Equation (2):

(2) $$\left[\begin{array}{c} x_{i}\\ y_{i}\\ 1 \end{array}\right]=\left[\begin{array}{ccc} f_{x} & \gamma & c_{x}\\ 0 & f_{y} & c_{y}\\ 0 & 0 & 1 \end{array}\right]\left[\begin{array}{ccc} r_{00} & r_{01} & r_{02}\\ r_{10} & r_{11} & r_{12}\\ r_{20} & r_{21} & r_{22} \end{array}\left|\begin{array}{c} t_{x}\\ t_{y}\\ t_{z} \end{array}\right.\right]\left[\begin{array}{c} x_{w}\\ \begin{array}{c} y_{w}\\ \begin{array}{c} z_{w}\\ 1 \end{array} \end{array} \end{array}\right]$$

where

*f*_*x*_ and *f*_*y*_ are focal lengths;
*c*_*x*_ and *c*_*y*_ are center-point coordinates of the image (principal point);
*γ* is axis skew (usually assumed 0).

The [**R**|**t**] matrix is usually extended into a single 4 × 4 matrix for the sake of convenience—as seen in Equation (3). This matrix allows one to project points from the world to camera space (coordinate system), and, thus, is sometimes referred to as world to camera, world to view, or simply view matrix.

(3) $$\left[R|t\right]=\left[\begin{array}{ccc} r_{00} & r_{01} & \begin{array}{cc} r_{02} & t_{x} \end{array}\\ r_{10} & r_{11} & \begin{array}{cc} r_{12} & t_{y} \end{array}\\ \begin{array}{c} r_{20}\\ 0 \end{array} & \begin{array}{c} r_{21}\\ 0 \end{array} & \begin{array}{cc} \begin{array}{c} r_{22}\\ 0 \end{array} & \begin{array}{c} t_{z}\\ 1 \end{array} \end{array} \end{array}\right]$$

The matrix of intrinsic camera parameters **K** represents the transformation of a point from camera to screen (or, alternatively, image) space. The matrix can be assembled from known camera parameters, such as resolution and field of view or focal lengths (more on that later). By plugging image points (2D) and corresponding world points (3D) into Equation (2), it is possible to compute rotation and translation vectors, and therefore to construct the world to camera or view matrix (3), which can be used to transform points from world into camera space.

In the new implementation, the scene model is stored in a JSON (JavaScript Object Notation) configuration file, which contains all other positions of cameras and a list of boundaries with their corresponding names and displayed colors. The boundaries are stored as a list of vertices that are connected one by one. The mapping points are then used to calculate the screen to world transformation matrix for every camera when the scene is loaded. The transformation matrix is then used to project detected objects into the scene model.

The result of the scene calibration is a configuration file of a scene model in a single-coordinate system with cameras with known projection matrices. A calibrated camera shows its field of view using visible arms on the ground plan. With the screen to projection matrices, it is possible to assign a line in the same 3D coordinate system to each image pixel of every camera. These lines are then used for localizing detected people and placing them into the scene. In order to model a multi-room exposition or any larger complex environment with multiple cameras, the scene abstraction had to be rewritten almost from scratch and improved significantly.

## 3. Results

The simple detector—YOLO pre-trained for thermal tasks based on the FLIR thermal dataset was used. The approach required us to redefine the parametrization of the detection. Every image is split into a grid, where each grid-cell is responsible for predicting several bounding boxes and confidence scores for each bounding box. It specifies how sure the detector is that a certain bounding box actually contains an object, and if there is an object, what kind of object it is. For training the YOLO object-detection models, the official YOLOv4 Darknet implementation was chosen. YOLOv4 is superior to all other object detectors in terms of both speed and accuracy.

There are currently no pre-trained YOLO models for thermal data. The only way to utilize the YOLO object detector to detect people in thermal images is to create a custom, annotated, thermal dataset and use it to retrain a YOLO detection model which has been originally trained on a large COCO image dataset. In other words, an extensive thermal image set had to be captured and manually annotated. YOLOv4 documentation suggests having at least 2000 images in the custom dataset for each class we want to detect. In order to construct the custom thermal dataset, two different thermal datasets were combined.

The first one is the only publicly available thermal dataset from FLIR and the second one was created using previously constructed thermal units with thermal cameras. Since the trained model would be used exclusively with the low-cost Lepton 3.5 camera module, it made sense to combine the FLIR’s thermal dataset with one captured using the Lepton 3.5 camera. This way, it was possible to harvest features specific to the Lepton 3.5 and also the essential characteristics of people on generic high-resolution thermal images. The FLIR’s thermal dataset includes over 14,000 thermal images with five annotated classes in raw Y16 and normalized gray-scale RGB888 formats. Only thermal images containing people were selected.

The FLIR’s dataset uses the COCO annotation format to store object annotations for images. This format is not supported by the YOLO implementation that is used in this project, and, therefore, the annotations had to be converted into the YOLO format using a custom script. The Darknet framework comes with pre-trained YOLO models which have been trained on the COCO image dataset to detect up to 80 classes from regular color images. A YOLO model cannot be used for thermal data straight away for obvious reasons. On the other hand, it is also not necessary to train the YOLO model from scratch, which would require a huge dataset, computational resources, and plenty of time. In this case, the pre-trained model can be retrained and repurposed for a specific task—that is, to detect person-objects in thermal images.

Even though the YOLO pre-trained model has been trained on a color dataset, it has been proven that it can handle gray-scale images as well. The model is fed by gray-scale images with three channels, meaning that each channel of every pixel has the same eight-bit value. Merging the FLIR’s and the custom thermal dataset resulted in 13,416 thermal image files with 53,628 annotated person-objects. This amount is significantly more than the minimum of 2000 suggested by the YOLOv4 manual, which is a good predisposition for training a rigid and reliable object detector.

**Figure 5:**
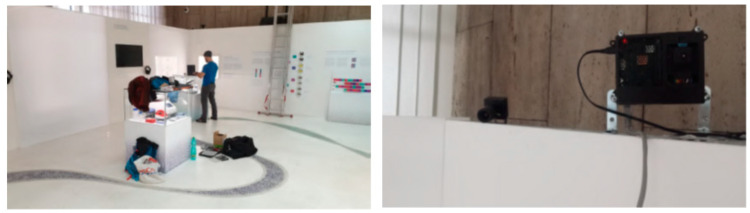
Illustration of the thermal unit installation in the National Museum, Prague [46].

Within the scope of the project, a total of three thermal units were assembled and deployed in two large exhibition halls. The new detection and localization system was used to construct a heatmap of the exposition revealing the most favorite areas. The thermal units were installed in the National Museum, Prague to overwatch an ongoing exhibition with the aim of extracting information about the visitors’ movement [46]. More specifically, the outcome of utilizing thermal units at this stage was to extract heatmaps corresponding to visitors’ common whereabouts. The heatmap can potentially yield hidden information about visitors’ behavior in specific parts of the exhibition, which is very valuable information for the museum’s creative department to know how to ergonomically build the best-suitable exhibitions for visitors. Figure 5 demonstrates the installation process in the museum.

**Figure 6:**
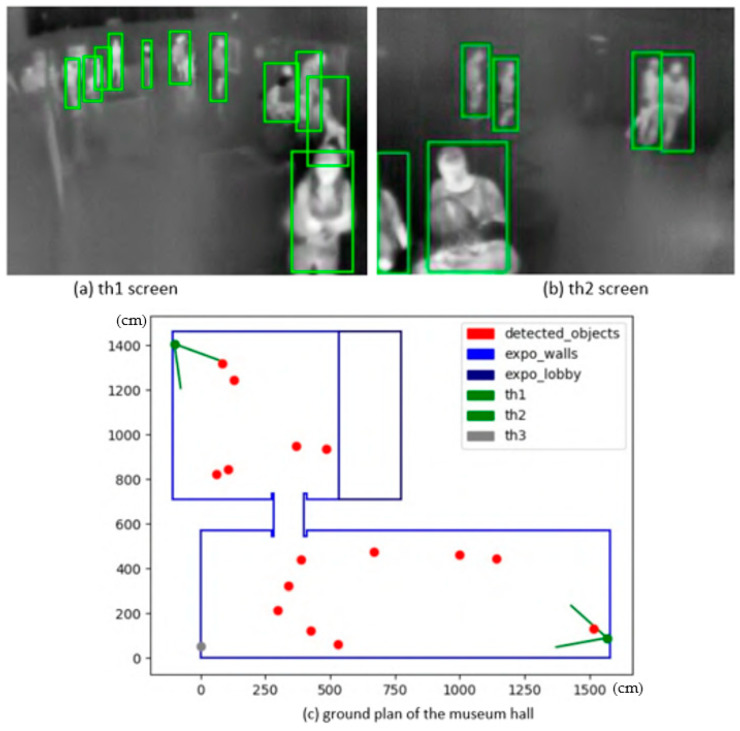
Demonstration of the live detector and locator tool in action: (**a**) screen of camera 1; (**b**) screen of camera 2; and (**c**) ground plan of the museum hall. Blue and green rectangles show the detected persons in the scene. The green ones are much better, because they differentiate nearly every person from each other, in comparison to the blue one, where groups of people are joined together.

The scene abstraction, according to the configuration file supplied, calibrates all cameras with defined mapping points, connects to their live feed, displays the view from each camera in a separate window with the detector running, and plots every detected object into the ground plan. The ground plan is updated with every frame, again, in a separate window. The first stage was a simple detector based on scene imaging, and then the following step was towards the YOLO real-time system. The live detector and locator in action with two active cameras and the merged locations of detected people are demonstrated in Figure 6.

**Figure 7:**
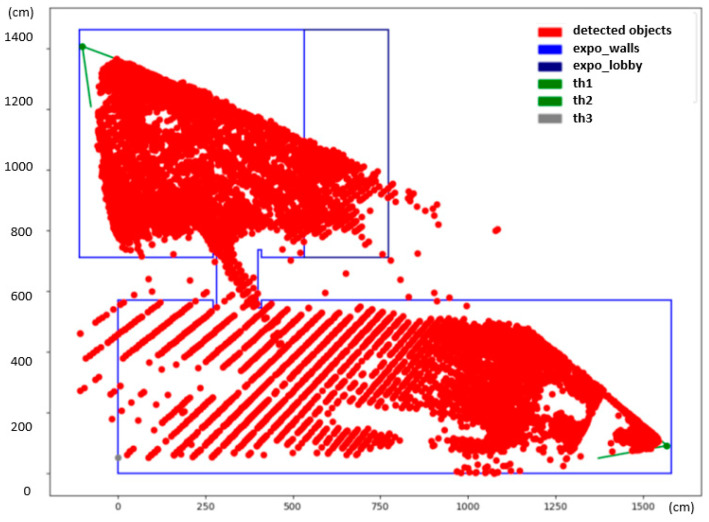

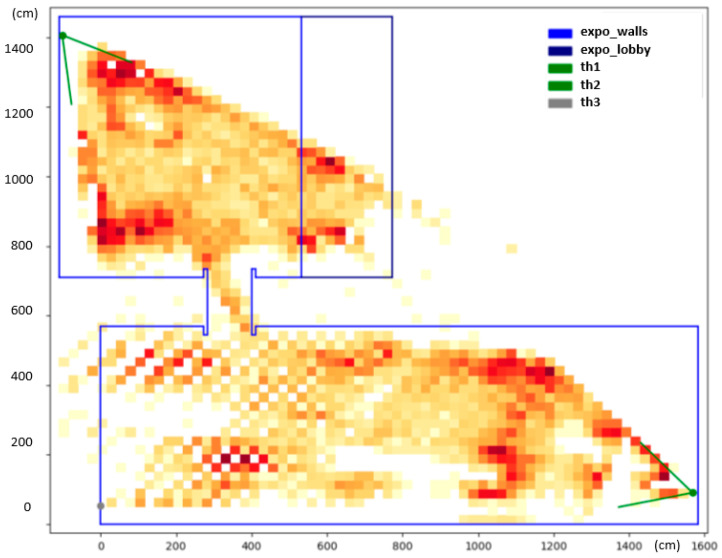
Heatmap illustration constructed with the custom YOLO thermal detector.

The final heatmap is depicted in Figure 7. The heatmap reveals interesting information about the common whereabouts of people. The dark-red color shows spots in the exposition that were occupied by people more often. These hotspots can be associated with specific parts of the exhibition that were particularly interesting to the visitors. The white spots in the middle of the larger hall correspond with no or a very small amount of detections, and, in fact, these areas were obstructed by show-cases and panels.

It is, however, noticeable that there are larger gaps between detected positions further away from the camera in the lower hall. This has to do with the small resolution of the camera, as mentioned previously. One pixel change in the camera view can translate up to a one-meter-large step in the scene, which introduces artifacts into the heatmap. No person can ever be detected in the white regions of the checkerboard pattern, visible on the bottom left side of the heatmap, as they correspond with positions in between two pixels in the thermal image. There was a third camera installed on the opposite side of the exhibition hall (th2, marked gray) that would eliminate this problem; however, not long after the deployment, the camera went offline and could not be revived before the end of the exhibition.

The museum represents the ultimate challenging environment for a detection and localization system that uses a Lepton camera with relatively small resolution. The modeled scene is large and contains many people. If a smaller room is considered with fewer people and, therefore, not so many occlusions, with the new YOLO detector, the new system would not have any problems and would yield reasonably accurate positions at all times.

Reflection of a part of the IR radiation on the glass was a problem. As a standard camera and also the detector would classify a mirror image of a person as a real person, the same thing also happens in thermal imagery, only it is a bit more unpredictable because the mirrored person is not acquired in the visible range, i.e. thermal reflections need to be taken into account. Usually, flat and shiny surfaces or, for example, a polished floor, reflect infrared radiation very well.

These issues make a simple comparable thermal detector unusable for the project, as one of the requirements is being able to detect individual persons in a larger area with more than a few people, for example in a museum exhibition hall. Therefore, a new detector based on a completely different method was chosen, designed, and implemented to suit the needs of the project. The YOLOv4 detector produces surprisingly good results, even for a very complex scenes that pose a real challenge for the thermal imaging of people.

## 4. Discussion

The goal of the project was to utilize small low-cost thermal imaging cameras to create a system that solves the problem of detecting and locating people. This can find applications in many areas, like hazardous area guarding, security systems, people-flow analysis for marketing purposes, and others. The advantage of using a system based on thermal imaging is that lighting conditions are irrelevant for correct functioning. The system may be deployed in locations where privacy plays an important role because the camera resolution and thermal imagery, in principle, prevent facial recognition, yet allow for detecting people. It is an easily deployable system supporting large complex scenes with multiple thermal cameras. In order to manage the camera remotely, a custom power switch circuit was designed, allowing one to turn the camera on or off remotely using a GPIO pin on the Raspberry Pi. The printed circuit board with the control circuit placed in between the camera and the Raspberry Pi enables the camera to be turned on and off remotely, forcing a full reboot that solves the problem of freezing up.

The Lepton 3.5 together with the Raspberry Pi 3B+ and the custom-printed circuit board were placed and encapsulated in a custom 3D-printed enclosure box, which represents a single thermal unit. The thermal unit can be safely transported, presented, or deployed to a new environment. Since it is expected that more thermal units will be assembled in the future, the process of configuring each thermal unit has been automated using the Ansible tool. A custom Ansible playbook was created, taking care of all installation steps, libraries, and dependencies. The Ansible playbook is used to prepare each new thermal unit. With the upgrade of the camera, new commands had to be implemented in the control software in order to be able to utilize the new features of the camera.

At this occasion, the control script was written. A total of 38 commands with all methods have been added to the control tool. The capture library was designed according to the client–server model with built-in data transfer via TCP sockets. The thermal unit now runs a server implementation written in C++ and is sped-up with dual-segment and dual-frame buffering. When a client connects to the server, it starts pulling raw thermal frames from the camera and sends them directly through the open TCP connection or, optionally, through a zlib compressor beforehand.

As a part of the new v4l2lepton3 capture library, two clients have been implemented. The first one in C++ preserves the original usage of the virtual video device, allowing for a remote thermal unit to be connected as a local video device. The second client, implemented in Python, can be used on its own to display a live thermal feed, but more importantly, it is used in the rest of the detection system as it is very simple and easy to include, yet sufficiently fast.

The merged thermal dataset was used to train several different YOLO object detectors with different network sizes, including the recently released state-of-the-art YOLOv4 real-time object detector based on a deep neural network. From the trained models, YOLOv4-320 was chosen to be used in the final detection system, as it performed the best. For testing thermal images and also on a real-time thermal video, the detector performs incomparably better than a comparative detector (a simple one based on filtering temperatures outside the human-body-temperature range and image processing), and is able to reliably detect a dozen people regardless of their pose in a complex scene with partial occlusions. The camera used in the comparative version was the Lepton 3, which does not have the true radiometry feature, so it was necessary to provide the mapping function between incident flux and real temperature. This function has been only an approximation with a relatively large error.

**Figure 8:**
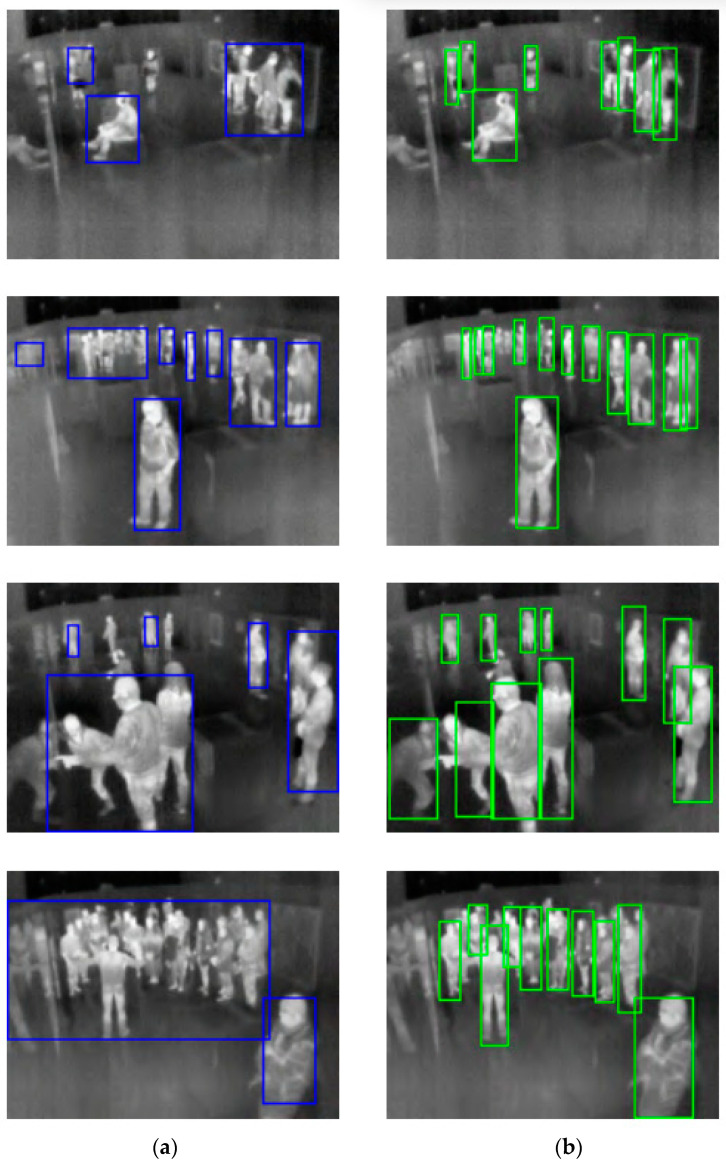
Detector differences. (**a**) Comparative thermal detector, (**b**) YOLOv4 thermal detector.

When compared with object detection models, YOLOv4 is the state-of-the-art real-time detector. The custom YOLO thermal detector is fast, has a remarkable accuracy, and solves almost all problems that the comparable thermal detector based on trivial image processing had. It deals well with larger groups of people and occlusions, and eliminates false detections of warm objects like radiators, TVs, and so on. The thermal reflections are, however, still problematic, and there is no easy fix for that. The YOLOv4 detector does a surprisingly good job even for very complex scenes that pose a real challenge even to humans. Differences between the YOLOv4 detector and the comparable one are shown in Figure 8.

Everything the detector needs is a single 160 × 120 thermal image enhanced by temperature filtering and contrast-limited adaptive histogram equalization (CLAHE). Even though the new YOLOv4 detector is significantly slower than the comparative detector, it is still faster than any other currently available object detector based on a neural network and fast enough to process a thermal video at full speed (at 8.7 Hz) without decreasing the frame rate.

The mathematics behind reverse-projecting image points into a 3D scene model stayed the same; however, the scene-abstraction software was redone from scratch. The scene is stored in a JSON configuration file and supports multiple cameras and multiple polygonal boundaries with different colors and names. The scene-abstraction software also includes a visual camera-calibrator tool, which simplifies the calibration process of every newly installed camera.

The tool connects to a camera, displays its thermal feed in real time, then the user selects significant points in the thermal image and enters the corresponding real-world coordinates of those points. The mapping coordinates are then stored in the configuration file of the scene and used every time the scene is loaded to compute the projection matrix of each configured camera. The project also contains plenty of supporting scripts that can be used for testing or troubleshooting the system. This includes a script for disconnecting the camera completely, for turning it back on, the Python v4l2lepton3 client implementation with the detection performed on every frame with the option to save the frame, scripts for single-frame capturing (used with scheduling locally and remotely), a script for running the detector on raw thermal images from a specified directory, and so on.

The complete system is implemented in the detect_live.py file, which loads the preconfigured and calibrated scene, connects to every calibrated camera in the scene, shows their live feed in separate windows, and draws locations of detected people in the ground plan of the observed scene. The system is in agreement with comparable tracking thermal imaging [47]. Within the scope of the cooperation with the National Museum in Prague, the detector has been applied to the collected thermal dataset with the aim of constructing a heatmap of visitors’ frequent occurrence within the exhibition.

## 5. Conclusions

The final result of the presented project is an easily deployable person-detection and -localization system based on thermal imaging that supports multiple cameras and can serve as an input for other systems that take actions by knowing the positions of people in monitored environments. This includes alerting security staff, analyzing the flow of people for marketing purposes, and controlling the environment in working places or smart homes. The system can also be used for thermal monitoring outdoors.

The thermal unit was designed with a custom enclosure box, a new camera, a custom control circuit, and a host computer. The main contribution of the system is in its application. There are currently no pre-trained YOLO models for thermal data. The only way to utilize the YOLO object detector to detect people in thermal images is to create a custom, annotated, thermal dataset and use it to retrain a YOLO detection model which has been originally trained on a large image dataset. The process of configuring the thermal unit was automated using Ansible. The v4l2lepton3 control and capture library was designed from scratch. The control part supports many more commands with all methods and translated options. The capture part was split into two parts—a server and a client. The C++ multithreaded implementation of the server was sped-up using double-segment buffering, double-frame buffering, and a reduced number of system calls.

It does not lose synchronization with the camera. It can recover from any kind of error and allows for zlib compression. There are two client implementations available. The C++ one uses a virtual video device to bring the remote thermal feed into the local machine for generic processing; the Python implementation is simple and easy to use or include in other projects. It is used in the detection software, but can also be used for quick previews. The scene-abstraction software has been redesigned so that, now, a scene is abstracted in a JSON configuration file and supports multiple polygonal boundaries and multiple cameras, which can be calibrated visually using a visual calibrator tool.

Finally, the comparative thermal detector showing poor results in larger scenes with more people has been replaced by the new state-of-the-art YOLOv4 real-time object detector trained on a custom thermal dataset that was created by merging the FLIR’s public thermal dataset and a custom one created in the Czech National Museum within the scope of an ongoing cooperation. The new detector is far superior to the comparative detector and can reliably detect people even in some of the most challenging situations.

The final detection system loads a preconfigured scene, connects to all cameras, displays their real-time thermal feeds, and, after the detection is performed, the detected persons are marked in the ground plan representation of the scene. The new detector was applied to the captured data from the National Museum with the aim of constructing a heatmap of visitors’ behavior. The built heatmap proves the capabilities of the detection system and may be beneficial for building new exhibitions in the museum in the future.

By having the new detector, the process of estimating the locations of detected objects from their bounding boxes becomes an area for possible improvement. The accuracy of an estimated location rapidly decreases with distance because of the low resolution of the thermal camera. About 16 m away from the camera, a difference of one image pixel can easily translate into a 1 m difference in the scene model. If a bounding box around a person is moved even by a few image pixels, its estimated location can change significantly.

In order to reverse-project an image point into a single point in the scene model, it is necessary to provide some additional information—for example, the *z* coordinate of the searched point. For reverse-projecting the feet of a detected person, the *z* coordinate would be set to 0; alternatively, 170 cm would be used for the head position (to represent an average person’s height). Using the head position is usually less accurate than the feet, as the height of people varies naturally. The position of feet works well when the camera is located high above ground or there are not many people in the scene. If neither condition is met, there is a higher possibility that a person would have their feet occluded by a different object.

In that case, the system would assume that the person is further away and misplace him/her completely. When a bounding box is touching the bottom of an image, the system expects that the feet of the detected person are not visible and uses the head position instead. This, however, does not solve the issue with occlusions.

One solution might be to train the detector to detect two classes—a torso and a whole person. That would require reannotating the whole dataset and longer training with unsure results, because the detector would then detect both the torso and the whole person and the system would have to identify that those two detections belong to the same person, which adds more room for error.

Both problems could be solved by adding another camera to observe the same scene from a different angle. The camera would have a priority to localize objects closer to it and both cameras could agree on the same objects. The additional coordinate required for placing the detected object into the scene would be provided from the two cameras using stereo vision. This feature shall remain on the top of the list of future upgrades.

Another possible improvement could be implementing allowed and blocked area concepts for localization. In the current implementation, there are no rules saying which section of the scene is marked for the possible occurrence of people and there is no way to tell which part of the scene is actually observable from which camera. By being able to determine which area of the scene is observable, it would become possible to exclude incorrect locations of detected people that lie outside the observable part of the scene. These outliers are often caused by thermal reflections, large occlusions, or the small resolution of the camera.

The future improvements might also tackle the lens distortion of Lepton cameras, as it becomes apparent for some particular modules. Another interesting feature to implement in the system could be person tracking. Each detected person would obtain an ID, and their movement through the scene would be stored in a database. This kind of data could be used for a more specific type of people-flow analysis where we could, for example, calculate the most typical direction of the movement of people. The candidate technology for object tracking could be the new DeepSORT [48]. The development of the system could be for 3D thermograms with a porTable 3D measurement system based on geometric calibration and data structure adaptation [49].

## Data Availability

Not applicable.
